# Are Skeletal Muscle Changes during Prolonged Space Flights Similar to Those Experienced by Frail and Sarcopenic Older Adults?

**DOI:** 10.3390/life12122139

**Published:** 2022-12-19

**Authors:** Alessandro Cannavo, Angelica Carandina, Graziamaria Corbi, Eleonora Tobaldini, Nicola Montano, Beatrice Arosio

**Affiliations:** 1 Department of Translational Medical Sciences, Federico II University of Naples, 80131 Naples, Italy; 2 Department of Clinical Sciences and Community Health, University of Milan, 20122 Milan, Italy; 3 Department of Medicine and Health Sciences, University of Molise, 86100 Campobasso, Italy; 4 Department of Internal Medicine, Fondazione IRCCS Ca’ Granda, Ospedale Maggiore Policlinico, 20122 Milan, Italy

**Keywords:** sarcopenia, frailty, aging, space flight, microgravity

## Abstract

Microgravity exposure causes several physiological and psychosocial alterations that challenge astronauts’ health during space flight. Notably, many of these changes are mostly related to physical inactivity influencing different functional systems and organ biology, in particular the musculoskeletal system, dramatically resulting in aging-like phenotypes, such as those occurring in older persons on Earth. In this sense, sarcopenia, a syndrome characterized by the loss in muscle mass and strength due to skeletal muscle unloading, is undoubtedly one of the most critical aging-like adverse effects of microgravity and a prevalent problem in the geriatric population, still awaiting effective countermeasures. Therefore, there is an urgent demand to identify clinically relevant biological markers and to underline molecular mechanisms behind these effects that are still poorly understood. From this perspective, a lesson from Geroscience may help tailor interventions to counteract the adverse effects of microgravity. For instance, decades of studies in the field have demonstrated that in the older people, the clinical picture of sarcopenia remarkably overlaps (from a clinical and biological point of view) with that of frailty, primarily when referred to the physical function domain. Based on this premise, here we provide a deeper understanding of the biological mechanisms of sarcopenia and frailty, which in aging are often considered together, and how these converge with those observed in astronauts after space flight.

## 1. Introduction

After long-term space flight, astronauts present with health problems with multisystemic dysfunction [1,2,3]. In addition to microgravity, prolonged space missions involve several environmental and operational stressors (e.g., decompression, dietary restrictions, psychological factors related to high workload under pressure, operational and interpersonal distress, isolation, and confinement) that lead to an impairment in the physiological reserve. Among the systems involved, the musculoskeletal system is one of the most affected as the disuse and unloading of muscles in microgravity lead to significant atrophy [4]. Importantly, this form of microgravity-induced muscle atrophy is problematic for its fast development and severity, with muscle mass diminished by up to 20% after a 2-week space flight or up to 30% after longer missions (3–6 months) [5]. In addition, similar to the effects observed in the older people, this skeletal muscle atrophy may induce adverse effects systemically impacting the cardiovascular and nervous systems [6,7]. For instance, a common problem of orthostatic intolerance has been observed in both astronauts and hospitalized aged patients [8,9,10,11]. Together, these problems can hamper or preclude astronauts’ mission tasks, thus demanding more investigations aiming to identify the molecular mechanisms responsible for these microgravity-dependent effects along with clinically relevant biological markers that will allow the design of tailored countermeasures. Of note, the current knowledge around the aging process can be exploited better to understand most of the mechanisms behind space flight-induced physiological modifications, as Nandu Gowsmani [8] stated in the exciting review: “Geriatrics meets Spaceflight!”. In this regard, studies around sarcopenia, a syndrome characterized by a loss of muscle mass and function in older individuals, have been proposed as an analog of the muscle loss observed after space travel. It is worth noting that sarcopenia is considered the biological substrate of frailty, a condition of the increased vulnerability to stressors that typically leads to adverse outcomes [8,12,13]. Hence, this review article will provide an update on the most recent clinical and experimental data on frailty and sarcopenia in older adults. Further, we will debate how the clinical and biological characteristics of this age-related syndrome are comparable to the physiological changes observed in astronauts after space flight [14].

## 2. Frailty and Sarcopenia during Aging

In the last century, the amount of people reaching old age has grown exponentially, with the number of people aged over 60 years old reaching almost 22% in 2050 [15]. Of course, prolonged life expectancy is accompanied by an increased risk of chronic degenerative diseases, frequently observed in older populations, with national healthcare systems encountering this evolution with huge costs [16,17]. For many years, it has been postulated that aging “per se” is the critical condition for the onset of many age-related diseases. However, the relationship between aging and age-related diseases is likely much more complex, since aging is the major risk factor for these diseases, and common biological mechanisms are shared among them [18]. It is believed that the deviation from healthy aging to the disease’s onset depends on the rate of cellular and molecular processes implying that aging and age-related diseases are two different trajectories of the same process [18]. This determines that the courses of aging are different among individuals and that chronological aging is very different from the biological one. Furthermore, the interactions between genetic profile, environment, and lifestyle affect the individual’s ability to adapt to the various changes occurring over time [19,20]. Overall, the physiological changes that portray old people are the results of each individual’s adaptive strategies from a biological point of view [21], balancing the physiological decline that occurs during aging. In this context, critical events can immediately precipitate the response-ability, intended as the physiological reserve of the individual, thus modifying the aging trajectory [22]. This higher clinical complexity is well represented by the concept of frailty, a condition characterized by the increased vulnerability to stressors and reduced homeostatic reserves [23]. From a biological point of view, frailty is driven by the gradual, lifelong accumulation of molecular and cellular defects that involve different organs and systems (e.g., skeletal muscle, brain, respiratory, cardiovascular, and endocrine systems) [23]. Indeed, frailty is highly prevalent in the general population (~15%) [24]. Under these perspectives, frailty is indicated as a promising way of capturing the physiological decline, as well as the biological aging of the individuals [25]. Although there is a general agreement on the theoretical definition of frailty, the clinical identification is difficult due to pathophysiological complexity and clinical manifestations that lead each person to experience different degrees of “fragilization” [26,27,28,29]. Moreover, frailty is an operational definition used to describe a clinical condition measured with different constructs, and for this reason, incorrect interpretations are created [30]. There are multiple definitions to quantify frailty [30], and many operational approaches have been proposed over time [31], generating a relevant problem and reducing the ability to predict adverse outcomes if we consider that the subtle fluctuations of frailty are very difficult to detect [32]. Predominantly, the operational tools are based on two models: the frailty index (FI) [33] and the frailty phenotype (FP) [34]. The FI mirrors the biological age of the organism presenting the ratio of health deficits manifested by the individual at the end of a comprehensive geriatric assessment [25,35], demonstrating its applicability, even among long-lived people [36,37]. Furthermore, the FI is able to well capture the so-called “gender-paradox” [38], in which women have experienced greater longevity than men [39], albeit this survival advantage is linked to higher rates of disability and poor health during women’s lives [38].

In fact, physical decline is considered the cardinal sign of frailty [34,40], which motivates the fact that most frailty assessment tools are built on the FP [34]. Indeed, aging is typically characterized by muscle wasting that progressively causes disability, loss of muscle function and of self-sufficiency in older subjects. Muscle mass reaches its peak between 30 and 40 years of age and starts declining after that [41], up to a reduction of 25–30% in the cross-sectional area of the skeletal muscle and 40% in muscle strength [42]. This phenomenon is called “sarcopenia”, a term coined by Rosenberg at the end of the 1980s [43,44] to describe the age-related loss of muscle mass and later revised to include reduced muscle strength and/or function (i.e., dynapenia) [45,46,47,48,49]. At present, muscle weakness is the critical factor in diagnosing people with sarcopenia and in clinical decision-making [49]. As a result, sarcopenia recently received a specific International Classification of Diseases, Tenth Revision (ICD-10) [50], making it a formally recognized disease [51]. The etiology of sarcopenia is multifactorial, involving many biological mechanisms [52,53], such as the neuromuscular junction dysfunction, reduced satellite cell number/function, intramuscular adipose tissue infiltration [54], as well as chronic inflammation [55]. 

Indeed, the involvement of neurological factors in the etiology of sarcopenia has been previously reported [56]. It is noteworthy that the PF model shows substantial overlaps with sarcopenia, since both cause a physically inactive lifestyle and fatigue [57]. Under this perspective, it has been proposed that sarcopenia may be the biological substrate for the development of physical frailty [57,58]. However, the causal relationship between the two manifestations remains largely unknown. For this reason, recently it has been coined the term ”physical frailty and sarcopenia” to merge the two conditions into a single entity [59] in which sarcopenia is intended as the biological substratum of physical frailty [57]. This concept is particularly applicable in older people whose ability to regulate the musculoskeletal system and cope with stress lose much of their efficiency [60], causing multisystem dysregulations.

To further complicate the picture, skeletal muscle can function as both an endocrine and a paracrine organ through the secretion of mediators, such as myokines, that bidirectionally link muscle to skeletal tissue [61,62].

## 3. Biological Mechanisms Underlying Sarcopenia and Frailty

The pathogenesis of frailty and sarcopenia in older people is suggested to encompass multiple biological systems [23,63,64,65]. In this regard, these two syndromes share several common risk factors, such as immune and inflammatory responses, hormonal dysregulation, and oxidative stress [66]. In this complex scenario, the mitochondrial dysfunction in skeletal myocytes is recognized as a major driver of sarcopenia. Moreover, the contribution of the systemic processes (e.g., inflammation, hormones) to the muscle mitochondrial dysfunction remains to be fully elucidated.

Given this, frailty and sarcopenia are considered highly interrelated [66,67]. This section provides a brief overview of the current understanding of the key pathophysiologic processes of each of these conditions.

### 3.1. Immune Activation and Inflammation 

A persistent immune system activation and a heightened inflammatory state are undoubtedly the most prominent and documented typical hallmarks of advanced age and a major contributor to several age-related pathologies, including frailty and sarcopenia [68,69]. This inflammatory process, identified with the term of “inflammaging” is often facilitated by physiologic and pathophysiologic alterations of the immune system occurring with aging, such as “immunosenescence”, an impairment of the functionality of immune cells that contributes to an increased incidence and severity of infections in older subjects [70,71]. 

In this context, a critical role is played by IL-6, a pro-inflammatory cytokine, whose age-related increased levels are well-known predictors of several pathophysiologic processes, including sarcopenia, physical disability, and motor performance decline even in well-functioning older adults (both men and women) [72,73,74,75,76,77,78,79,80]. 

In addition, a report from Leng et al. [81] provided the first evidence of a link between frailty and inflammation, demonstrating that community-dwelling older frail subjects presented with higher serum IL-6 levels than their non-frail-counterparts. To date, several studies have corroborated that this cytokine is directly related to frailty in community-dwelling older adults and in hospitalized people [82,83,84,85,86,87] and its secretion appears to be driven by an altered functionality of the immune cells (i.e., immunosenescence) in response to a chronic infection. Indeed, as demonstrated by Leng and colleagues [88], the peripheral blood mononuclear cells (PBMC) from older frail adults, after continuous exposure to bacterial lipopolysaccharide (LPS), proliferate less and augment the release of this cytokine. In line with these reports, Qu and coworkers [89] proved that stimulation with LPS of monocytes isolated from frail older individuals resulted in a more robust expression of genes encoding for chemokines and cytokines than their non-frail counterparts. Interestingly, in another report, Schmaltz et al. [90] demonstrated that a chronic cytomegalovirus (CMV) infection was significantly associated with physical frailty and that IL-6 enhances the magnitude of such association. Finally, in a recent report from Kawamura et al. [91], it has been demonstrated that the chronic exposure of mice to porphyromonas gingivalis (Pg) LPS (LPS-Pg), one of the major pathogenic factors for periodontitis [92,93], increases muscle atrophy participating to the development of sarcopenia. Of note, periodontitis is a chronic inflammatory disorder triggered by Pg and other periodontal pathogens, that colonize the periodontium and, thanks to its virulence factors (including LPS-Pg), stimulates the production of inflammatory mediators and cytokines. Periodontal pathogens can destroy the epithelium of the periodontal pocket, thus allowing the entry of noxious endotoxins and exotoxins into the bloodstream, a process that leads to bacterial dissemination and systemic infection, with a consequent rise in the inflammatory response. 

Importantly, as suggested by several studies, the fate of muscles in older subjects depends mostly by the severity and chronicity of inflammation [94,95]. In support of this proposal, Greiwe et al. provided data in humans suggesting that systemic inflammation (e.g., via the augmented circulating tumor necrosis factor alpha [TNF-α] levels) contributes to age-associated muscle wasting. These data were corroborated by Crossland and colleagues [96], who demonstrated that the LPS infusion in rats induced a significant systemic inflammatory response, accompanied with an increased expression of IL-6 and TNF-α in skeletal muscle, causing the loss of muscle mass and strength. In a human perspective, a recent report by Kamper and colleagues [97], using data from the Copenhagen Sarcopenia Study [98], observed that, during aging, the systemic levels of TNF-α and the C-reactive protein (CRP) increase especially in more physically frail older subjects, thus supporting the association between systemic inflammation and poor physical function. Of note, TNF-α plays a crucial role in the pathogenesis of sarcopenia and frailty, since it directly upregulates the nuclear factor kappa-light-chain-enhancer of the activated B cells (NF-κB) pathway, including the ubiquitin-proteasome system, thus leading to to the loss of skeletal muscle proteins and myofibrils degradation [99,100] and myogenesis inhibition [101].

### 3.2. Role of Myokines 

Skeletal muscles secrete many cytokines and factors called “myokines” with specific autocrine regulatory activities, including effects on the muscle metabolism, growth, and functionality, with effects also on inflammation and myogenesis [102,103,104]. Interestingly, such myokines may also systemically elicit paracrine functions on distant organs and tissues [97]. To date, several myokines have been identified, and some (described in this section) are relevant for their role in the pathogenesis of sarcopenia and frailty.

#### 3.2.1. Insulin-Like Growth Factor 1 (IGF-1)

IGF-1 has a robust stimulatory effect on protein synthesis in muscle cells. Therefore, this factor is a well-recognized regulator of the regenerative capacity of muscle fibers. Indeed, IGF-1 stimulates growth and proliferation and controls the cell differentiation in muscles, bone, and cartilage tissue. Lower serum levels of this myokine have been proposed as an index of frailty and sarcopenia in older adults [105,106]. For instance, lower serum IGF-1 levels are related to the diminished physical performance and handgrip strength [107,108], high risk of disability [109,110], and are independently related to the reduction of skeletal muscle mass [106] in older adults. 

#### 3.2.2. Myostatin

Myostatin, also known as the growth differentiation factor 8 (GDF8), is a muscle-derived protein and member of the transforming growth factor (TGF)-β superfamily. As IGF-1, myostatin is a myokine regulating skeletal muscle metabolism and muscle mass [111]. However, in contrast to IGF-1, myostatin negatively impacts skeletal muscle mass, enhancing proteolysis and inhibiting the protein synthesis [111]. In addition, myostatin has been demonstrated to partake in the process of skeletal muscle wasting, typical of aging. For these reasons, myostatin has been investigated for its potential involvement in sarcopenia and frailty [22,112,113]. In this regard, several studies (both clinical and experimental) have reported an association between high myostatin levels and low muscle mass [114,115]. However, the relationship of myostatin with these conditions remains highly debated and inconclusive [116]. Indeed, opposite findings [116,117,118] or a lack of association [119] between circulating myostatin and frailty/sarcopenia conditions have been reported. In addition, the activities and levels of myostatin appear to be differently modulated in older women and men. For instance, Bergen 3rd et al. [120], demonstrated that myostatin contributes to the higher prevalence of sarcopenia only in women. Conversely, Chew and colleagues [121], despite confirming the presence of such sex differences, showed that myostatin in men is a potential biomarker for coexistent sarcopenia and frailty in community-dwelling older adults. Moreover, myostatin changes are also dependent on age and comorbidities [118,122], thus claiming for further studies that define better the specific association between the levels of myostatin and frailty/sarcopenia-parameters.

#### 3.2.3. Irisin

Irisin, a peptide of 112-amino acids, is proteolytically cleaved and secreted from the fibronectin type III domain-containing protein 5 (FNDC5) [123]. Irisin is considered a vital myokine, primarily synthesized and secreted by the skeletal muscle following mild physical activity, and its levels appear to be associated with increased muscle mass and strength [124,125]. Indeed, at molecular levels, irisin is a positive regulator of the IGF-1 and mTOR pathways, enhancing the muscle protein synthesis [125,126]. Moreover, studies demonstrated that the irisin administration to human skeletal muscle cells increased IGF-1 and decreased the myostatin mRNA levels [125,127]. Importantly, irisin exerts anti-inflammatory effects and positively impacts the myotube glucose homeostasis [102,103]. Accordingly, irisin is considered a promising biomarker of sarcopenia and frailty [128,129] since its levels are markedly reduced during aging, and as demonstrated by Chang et al. [128], low circulating irisin levels are a sensitive marker for muscle weakness and atrophy.

#### 3.2.4. Follistatin

Follistatin is an endogenous inhibitor of the transforming growth factor (TGF)-β superfamily ligands, including myostatin, and thereby promotes the skeletal muscle hypertrophy [130,131,132]. Therefore, follistatin has been proposed as a potential therapeutic against muscle atrophy [133,134,135]. Further, despite the controversy, an association with frailty and sarcopenia has been provided in older adults for follistatin [119,136,137].

### 3.3. Vitamin D 

Nutritional factors play a significant role in the pathogenesis of frailty and sarcopenia [138]. Among these factors, Vitamin D and its deficiency have been demonstrated to affect the musculoskeletal function significantly [139]. Vitamin D is a fat-soluble vitamin primarily synthesized in the skin upon sunlight exposure (ultraviolet B rays [UVB]), and about only 10% is supplied by dietary intake [138]. Importantly, Bischoff-Ferrari et al. [140] demonstrated that aging was associated with the decreased intracellular vitamin D receptor (VDR) expression in human skeletal muscle tissue, and this was paired with a higher prevalence of a vitamin D deficiency. In addition, several observational studies provided an association between low vitamin D and sarcopenia and physical performance, in older adults [141,142], suggesting the involvement of the vitamin D/VDR system in muscle aging. Of note, the results from a study by Yu and colleagues [143], supported this thesis, demonstrating that a vitamin D deficiency can increase the incidence of age-related sarcopenia, by inducing oxidative stress, skeletal muscle senescence, and the senescence-associated secretory phenotype. Moreover, in a recent study [144], Parsanathan and colleagues showed that the co-supplementation of vitamin D and the antioxidant amino acid L-cysteine, in vitamin D-deficient mice, exerted beneficial effects on the skeletal muscle, improving the expression of the myogenic biomarkers and reducing the expression of the markers for musculoskeletal disorders, such as muscular dystrophy. Therefore, several randomized controlled trials have investigated the critical physiological role of this system within the muscle, demonstrating the beneficial effects of vitamin D supplementation on muscle function [145,146,147,148]. 

### 3.4. Oxidative Stress

It is widely accepted that a direct relationship between oxidative stress and aging exists [149,150]. Accordingly, in 1956 Denham Harman proposed, for the first time, the free radical theory of aging, where oxidatively changed cellular components progressively accumulate in the cells during the organisms’ lifespan, leading to a decline of the cellular functions [151]. This thesis has been corroborated by decades of studies in both animals and humans, and, importantly, among the tissues negatively affected by oxidative stress, the skeletal muscle is one of the most important, especially in older persons. Indeed, as age progresses, muscles exhibit increased levels of reactive oxygen species (ROS) and reactive nitrogen species (RNS) that, in turn, causes the oxidative damage of the biomolecules (e.g., oxidation of lipids, protein, and DNA; protein carbonylation; inhibition of the muscle cell differentiation; breakdown of the myogenic proteins, and damaged autophagy process) [152,153,154,155]. In addition, the accumulation of ROS with aging induces apoptotic signaling cascades [150,156,157], leading to age-related muscle loss [158,159]. Mitochondria are a primary source of ROS in skeletal muscle, and mitochondrial DNA (mtDNA) is especially sensitive to oxidative DNA damage [160,161]. Of note, mtDNA damage (i.e., deletion, mutation frequency, copy number) increases in human skeletal muscle with age and is associated with impaired physical performance and skeletal muscle atrophy [161,162,163,164,165,166,167,168]. Importantly, it was demonstrated how antioxidants, ROS, and antioxidant enzymes control, in a positive or negative manner, inflammation and macrophage polarization [169,170,171,172] a process that, in skeletal muscle, is particularly relevant since it regulates both tissue regeneration (after injury) and infection resolution [173]. Together, these mechanisms seemingly underlie the pathogenesis of sarcopenia and frailty [149,150,174,175,176,177,178]. Indeed, in 2007, Howard and colleagues [179] provided one of the first proofs of the importance of oxidative protein damage measurement in predicting muscle weakening in the elderly. In line with these data, in a cross-sectional study performed on older women, these authors found that protein carbonylation was independently associated with low grip strength in frail women, compared to their non-frail counterparts. Subsequently, Serviddio and coworkers [180] provided data about a direct association between oxidative imbalance and frailty. In detail, these authors found raised levels of oxidized glutathione (GSSG), malondialdehyde (MDA), and 4-hydroxy-2,3-nonenal-(4-HNE) protein adducts in the plasma of frail elderly patients (aged 65 and older), compared to non-frail patients. Analogously, Bellanti et al. [181] demonstrated that significantly greater blood GSSG and plasma MDA/HNE protein adducts were observed in sarcopenic, rather than in non-sarcopenic, elderly patients. For their part, Bernabeu-Wittel and colleagues [182] evaluated the association between oxidative stress marker levels (total antioxidant capacity to the reactive oxygen species [TAC-ROS] and superoxide dismutase [SOD]) with sarcopenia and/or frailty, discovering that these markers were enhanced in both the conditions and when these coexisted.

Despite several biological mechanisms that have been highlighted, further studies are needed to better define their causal relationship with frailty and the sarcopenia parameters and to identify new possible therapeutic targets.

## 4. Impact of Space Flight on Astronauts’ Skeletal Muscle Health

Microgravity associated with space flight, especially following prolonged missions, results in the substantial deconditioning of the musculoskeletal system [183,184,185,186,187,188] that can be exacerbated due to a negative energy balance [185,189,190,191] and by the mission duration [187,188]. Consequently, a substantial muscle mass reduction (atrophy) and an impairment of muscle strength and endurance capacity represent a serious medical problem for astronauts upon their return to Earth or during a long-duration space flight. In this sense there are many parallels between the effects of aging and space flight on the skeletal muscle function and structure that can be drawn. 

### 4.1. Clinical Manifestation of Sarcopenia/Frailty-like Phenotype in Astronauts 

The term sarcopenia (from Greek: “sarx” for flesh and “penia” for loss) was defined in 1989 by Irwin Rosenberg to generally describe an age-related loss of muscle mass and function [44]. However, it is clinically essential to specify that sarcopenia also alters the physical performance and function. Indeed, this condition is associated with frailty, a condition characterized by the reduced functional ability and increased postural instability, disability, and mortality [192]. For this reason, sarcopenia is considered a clinical analog for microgravity-induced muscle deconditioning observed in astronauts during short and long-term missions [193,194]. Importantly, due to disuse in microgravity and limited movement range, astronauts undergo well-characterized and described effects related to muscle tissue (wasting and/or atrophy) and the skeletal system (accelerated bone resorption) [179]. Atrophy is the main skeletal muscle feature associated with microgravity and is manifested as both losses of muscle size/ volume and reduction in the myofiber size [180].

Indeed, in astronauts, the antigravity muscles (e.g., soleus, gastrocnemius, quadriceps, and muscles of the back), that are typically used on Earth, are no longer utilized in the absence of gravity, thereby they remain in a typical state of unloading and disuse [195,196,197,198,199,200]. These changes induce alterations in the size of the muscle fibers, resting and active force, contractile velocity, and function of the neuromuscular junctions, causing substantial physical deficits, such as fatigue and decreased speed [8,201,202]. Indeed, the clinical and biological manifestations that astronauts manifest during and after space flight seem to resemble the clinical and biological characteristics of physical frailty experienced by older people [60,203,204].

It is well known that aging alters the skeletal muscle homeostasis, contributing to an imbalance between the muscle protein anabolic and catabolic pathways and leads to an overall loss of skeletal muscle [205]. For instance, muscle mass reaches its peak between 30 and 40 years of age and starts declining thereafter [41], with up to 50% of the mass being lost by the 8th decade of life [206]. This decline can rapidly progress in people less physically active and in acute or chronic conditions [207]. From a biological point of view, the muscle loss experienced by sarcopenic older patients is driven by the decline in the number of neuromuscular junctions, leading to a loss of size and number of muscle fibers (predominately type II) [208].

In addition, the instability of the neuromuscular junctions, together with the altered production of calcium during the excitation-contraction coupling of the muscle, appear to be key factors in the decline of muscle strength [209] and force production [210]. Moreover, muscle capillarization is critical in reducing the exercise capacity and sarcopenia onset [211], and in regulating the skeletal muscle maintenance [212,213]. Similarly, the interplay between the nervous system and skeletal muscles is a key factor in the pathogenesis of muscular atrophy, induced by prolonged space flight [202,214,215,216]. Indeed, much evidence indicates that the presynaptic modulation of motoneurons, from the spinal cord to the neuromuscular junction, may contribute to muscle atrophy associated with space flight [201]. 

Since muscle mass accounts for up to 60 percent of the body mass, pathological alterations in this tissue could have enormous consequences for astronauts. It has been shown that muscle fibers rapidly adapt to the space environment in terms of size, strength, metabolic properties, and vascularization [217]. The level of adaptation and recovery to the microgravity environment depends on the mission duration and on inter-individual differences. Pre-flight markers may be used to identify crewmembers at the most significant risk of an altered response to microgravity and unloading, and therefore to indicate the need for preventative measures [218].

Furthermore, sex/gender differences may impact the ability to recover and the adequacy of the metabolic response after a space flight. For instance, previous studies showed that female astronauts take longer to restore their metabolic balance during recovery [219].

Significantly, this effect may depend on the alteration in sex hormones (i.e., estrogen) which, concerning women, are grossly understudied in both space missions and simulated microgravity [220]. Indeed, previous studies have demonstrated that during the menopausal and post-menopausal periods, women present with an increased progressive muscle degeneration (i.e., decrease in the quality and muscle function) than their male counterparts, and this effect has been partly related to the reduction in estrogen levels [221]. In this regard, studies in rats that underwent space flight (7–14 days) demonstrated a decrease in oxytocin levels [222]. This hormone attenuates the hypothalamic–pituitary–adrenal (HPA) axes, dampens the stress responses in women, and indirectly correlates with the estrogens levels, since this sex hormone may increase the expression of oxytocin [223]. In addition, it should be kept in mind that other factors, such as impaired nutrition, hormonal dysregulations, and psychological stress, can trigger bone and muscle loss in older people and astronauts [180], accounting for the fact that there are very different aging phenotypes and different outcomes after the space flight [218,224].

Astronauts are subject to environmental stressors, such as isolation, a heavy workload, high noise and vibrations, exposure to radiation and toxins, limited nutrition, and the use of recycled air and water. These stressors could reduce their physiological reserve by altering their homeostasis and impacting the skeletal muscle function [201,202,225], as well as they can alter the biological mechanisms underlying aging [226,227]. There is growing evidence that the risk of frailty in older adults is strongly associated with an inadequate intake of food [228]. Indeed, a low dietary intake is one of the most critical factors of malnutrition responsible for the functional decline, physical frailty, sarcopenia, disability, and loss of independence [229,230]. In this regard, an inadequate caloric intake and altered protein content are also described in long-duration space flights affecting the astronauts’ metabolism and positive attitude [231].

Since some causal factors of sarcopenia, in the elderly population, are superimposable to the conditions to which the astronauts are exposed (e.g., food restriction, inactivity, social isolation), the assessment of pathophysiological mechanisms leading to muscle atrophy during space flight, could bring new insight on sarcopenia and frailty development.

### 4.2. Pathophysiological Mechanisms Activated in the Muscle by Prolonged Space Missions

**Figure 1:**
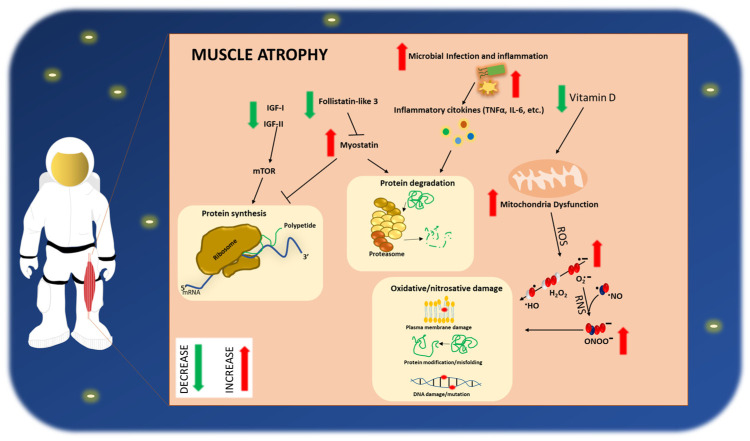
Schematic representation of the mechanisms leading to muscle atrophy in astronauts.

As discussed above, there is a keen interest in understanding the molecular mechanisms responsible for the muscle atrophy observed in astronauts during and after space missions (Figure 1). Notably, previous studies on rodents have demonstrated that most of the effects on the muscle structure and function, induced by space, are qualitatively similar to those found in humans, especially with aging [217,232,233,234,235,236]. The results of both short- and long-term missions showed that muscle undergoes a considerable mass reduction (about 20% in humans and 30–40% in rodents), compared to the ground controls. These effects are associated with an extensive gene expression rearrangement at molecular levels. For instance, Allen et al. [237] analyzed the expression of key genes involved in numerous cellular processes, including the cytoskeletal and mitochondrial functions, metabolism, cell cycle, and apoptosis, in the gastrocnemius of mice (space flight group) that were kept flowing on the mid-deck of the space shuttle Endeavour (STS-108/UF-1) for 11 days and 19 h. The authors’ analysis demonstrated that the mRNA levels of most of the genes analyzed were significantly altered by the space flight, compared to the controls (normal gravity). Importantly, these authors found a significant alteration in the PhosphatidylInositol 3-Kinase (PI3K)/Akt/mTOR pathway, which, as discussed above, represents critical regulator pathways of protein synthesis. In detail, an upregulation of the expressed genes involved in inhibiting this pathway, including the gene encoding for the PI3-kinase regulatory subunit p85α, which negatively impacts PI3-kinase signaling, has been observed. In addition, a robust increase in myostatin mRNA levels was found, which, along with the inhibition of the PI3K/Akt/mTOR pathway, supports the idea that space flight causes a molecular shift towards mechanisms that enhance protein degradation. In line with these findings, the authors observed a decrease in the mRNA levels of the myostatin binding/inhibiting protein gene follistatin-like 3 (FSTL3), underling a negative impact on the skeletal muscle mass. Finally, a significantly altered expression of mRNAs encoding for the TNF-α-induced protein 2 and Nfatc3 was observed. In line with these data, Lalani and colleagues [238] demonstrated that muscle atrophy in rats undergoing a 17-day space flight (NASA STS-90 NeuroLab) was associated with the upregulation of myostatin and the decreased IGF-II levels. Interestingly, these alterations were normalized upon restoration of normal gravity and caging conditions. Sandonà et al. [183] performed a long-term (91-days) experiment on mice (mice drawer system [MDS] program, sponsored by the Italian Space Agency onboard the International Space Station) demonstrating that this long-term exposure to microgravity is responsible for an impaired muscle mass associated with a reduced IGF-1 expression.

Along with myokines, many reports have demonstrated that a dysregulation in the immune system function occurs in rodents and humans immediately following short- and long-duration space flights with a shift toward inflammaging, which, as seen in the previous paragraph, is one of the main factors promoting frailty and sarcopenia [239]. Notably, such an alteration is mediated both by microgravity or by ionizing radiations [240] and mainly consists in changes in the leukocyte distribution, in the impaired function of immune cells, and in altered cytokine and inflammatory mediators’ production and release [239,241,242]. Moreover, studies in rodents have clearly demonstrated that this immune system dysregulation is responsible for an impaired ability of the host to respond to infections [243]. Indeed, astronauts face an increased risk for microbial infections because of the altered microbiome (dysbiosis) [244]. For instance, studies have reported that astronauts exhibit increased gingival inflammation and periodontitis [245]. Further, the latent viral reactivation has been commonly reported during space flight and represent a manifestation of the immune system dysregulation [246,247].

Finally, oxidative stress and the consequent damage related to the excessive production of ROS and RNS by the skeletal muscle, have been found in astronauts during space flight, that are responsible for the altered structural and functional integrities of this tissue [248].

Following space flight, a substantial deconditioning of the musculoskeletal system with consequent muscle atrophy is observed in astronauts. This effect is dependent on multiple factors, and among these, a reduction (GREEN arrow) or an increase (RED arrow) of the crucial factors involved in protein synthesis, degradation, and oxidative and nitrosative stress damage, have been described. In detail, it has been demonstrated that a reduction in the insulin growth factor (IGF-I or IGF-II)/mTOR (mechanistic target of rapamycin) system resulted in a reduction of the protein synthesis. Similarly, a decrease in follistatin-like 3 (FSTL3) with a consequent increase in myostatin levels, leads to the augmented protein degradation via the activation of the ubiquitin-proteasome system and to a parallel inhibition of the protein synthesis. Similarly, an increased concentration of pro-inflammatory cytokines, such as interleukin-6 (IL-6) and tumor necrosis factor-α (TNF-α), also stimulated by the microbial infections, resulted in the increased protein degradation. In addition to these mechanisms, a vitamin D deficiency is responsible for an increased mitochondrial dysfunction with a consequent increase in the reactive oxygen species (ROS), including superoxide anion (O_2_^·−^), hydrogen peroxide (H_2_O_2_) and hydroxyl radical (^·^HO). In addition, O_2_^·−^ can interact with nitric oxide radical (NO^·^), leading to the generation of the reactive nitrogen species (RNS) (e.g., peroxynitrite [ONOO^−^]). ROS and RNS cause oxidative damage to biomolecules (e.g., protein and DNA) with harmful effects on the skeletal muscle cells.

## 5. Potential Countermeasures

Based on current observations, aging-like physical frailty and sarcopenia conditions are observed in astronauts during and after space flight. Therefore, adequate countermeasures aiming at counteracting the adverse effects of space flight on astronauts can take into account the current strategies used to fight sarcopenia and frailty in older people. In this regard, physical activity and/or nutritional interventions are undoubtedly considered the forefront strategies to counteract muscle atrophy and bone mineral density in both older adults and astronauts [249]. In general, exercise training is mainly associated with systemically beneficial effects, positively affecting the skeletal muscles and other tissues/organs [250,251,252,253,254]. Among the effects reported on muscles, exercise has been shown to attenuate the imbalance between the muscle protein degradation and synthesis, reduce the oxidative damage and mitochondrial dysfunction, decrease inflammation, and stabilize the autophagy processes [252,253]. Interestingly, it has been described that a multicomponent intervention, based on physical activity with technological support and nutritional counselling, is associated with a reduction in the incidence of physical frailty and sarcopenia in older subjects [255].

In this context, exercise remains the primary countermeasure to mitigate the impairment in the physical performance that astronauts experience [256,257,258]. Indeed, recent studies have demonstrated that astronauts, using modern on-board resistive exercise devices, appear to be less susceptible to muscle changes [224,259]. However, despite the beneficial effects elicited by exercise training in astronauts, considerable muscle loss is still observed, thus demanding a period of rehabilitation upon their return to Earth [260,261]. In older adults, the effects of exercise are highly variable and mostly depend on the response to exercise (which is low in most subjects) and on the patients’ mobility in general [262]. For example, after resistance-type exercise training, the size increment of type II muscle fibers was mainly driven by individuals who had a higher muscle fiber capillarization at the baseline [263].

Therefore, other therapeutic approaches have been tested and implemented to counteract the effects of aging or space flight on skeletal muscle. In this regard, nutritional supplement to prevent a low vitamin D status, seemingly associated with muscle loss and impaired performance, has been adopted as an additional intervention and has been tested both in the geriatric population and in space flight studies. However, whether vitamin D supplementation in astronauts or old sarcopenic patients is beneficial or not in counteracting muscle atrophy, remains still controversial. Indeed, specific unresolved issues, including the complicated mechanisms underlying vitamin D activities on muscle tissue [264], the duration and dose of vitamin D supplementation need to be further investigated [148,265]. Finally, new drug candidates may find an ideal positioning, particularly among people that are non-responsive to lifestyle modifications because of the biological, clinical, and/or social factors [266,267]. Among these, myostatin antagonists (i.e., antibodies) have been extensively investigated under various clinical conditions associated with muscle loss and functional impairment. For instance, the anti-myostatin antibody (ATA 842) administration in elderly mice has been proven to increase muscle mass and strength [268]. In addition, in a multicenter study conducted in older people, it has been shown that myostatin antibody (LY2495655) improved the functional muscle power [269]. Of note, several myostatin pathway inhibitors are investigated in clinical trials for their potential impact on muscle atrophy [249], raising the possibility of using this therapeutic intervention also in astronauts and cosmonauts, to counteract long-term space flight-induced muscle alteration. In this sense, a study by Smith and coworkers [249] represents the first step towards implementing these drugs in astronauts, as they provide data in mice showing that the anti-myostatin antibody YN41 prevents space flight-induced atrophy. From these perspectives, it seems evident that combining pharmacological interventions with physical activity and nutritional support could be the gold standard to counter these adverse conditions both in space and on Earth.

## 6. Perspectives and Conclusions

**Figure 2:**
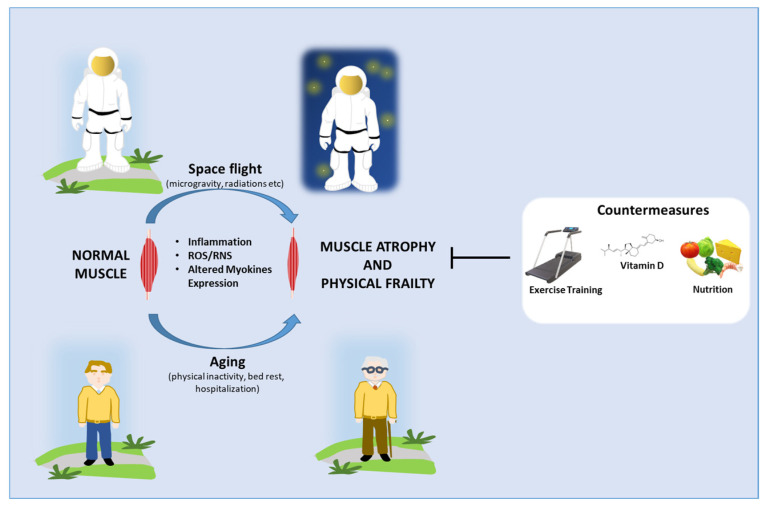
Schematic representation of the mechanisms leading to muscle atrophy and physical frailty in astronauts and older people and the potential countermeasures to fight these adverse conditions.

This review article summarized the current knowledge regarding how space flight affects the physical function of astronauts by altering skeletal muscle cells and function. The picture coming out from this analysis is that part of the harmful mechanisms activated in the muscles and systemically in astronauts are parallel to those observed in older people (see Paragraph 3 and Figure 2). Of course, further investigations on space flight induced effects and the recovery phase of astronauts are needed to clarify the main determinants and causes of the development of these aging-like pathological conditions with applicative consequences also in the geriatric field. In light of this premise, it is worth stressing that the convergence of Geroscience and gravitational/space research can significantly advance our understanding of human physiology and the biological mechanisms involved in adapting to stress.

Increased inflammation, reactive oxygen species (ROS)/R reactive nitrogen species (RNS), and the altered myokines expression have been found in astronauts and older people and are responsible for the altered structural and functional integrity of the skeletal muscle. Adequate countermeasures aiming at counteracting these adverse effects include exercise training, vitamin D, and multi-nutrient supplementation, have been shown to reduce oxidative damage and inflammation and stabilize the myokine expression.

## Data Availability

Not applicable.
